# BCGitis and BCGosis: Clinical Spectrum, Immunological Mechanisms, and Risk Management

**DOI:** 10.3390/vaccines13121179

**Published:** 2025-11-21

**Authors:** Qibin Liu, Xiyong Dai, Shuang Wei

**Affiliations:** 1 Wuhan Pulmonary Hospital, Wuhan Institute for Tuberculosis Control, Hubei Province Branch of National Center for Clinical Medicine of Infectious Diseases, Wuhan 430000, China; liuqibin0221@163.com; 2 National Engineering Research Center for Nanomedicine, College of Life Science and Technology, Huazhong University of Science and Technology, Wuhan 430074, China; 3 Research Institute of Pulmonary Infectious Diseases, Jianghan University, Wuhan 430000, China

**Keywords:** BCG vaccine, BCGosis, adverse events, immunodeficiency, newborn screening, risk management

## Abstract

Bacille Calmette-Guérin (BCG) remains the only licensed vaccine against tuberculosis (TB), administered to >100 million neonates annually. It confers approximately 70–80% protection against tuberculous meningitis and miliary TB in early childhood, under-pinning its continued use in high-burden settings. As a live-attenuated vaccine, however, BCG can rarely cause adverse reactions ranging from self-limited local lesions to life-threatening disseminated BCG disease (BCGosis), which almost exclusively occurs in infants with severe primary or acquired immunodeficiencies such as SCID, MSMD, CGD, or symptomatic HIV infection. Implementation of universal newborn screening for severe combined immunodeficiency (SCID) using the T-cell receptor excision circle (TREC) assay now enables prospective identification and deferral of these high-risk neonates, virtually eliminating fatal BCGosis. Here we synthesize global data published since 2010 on the clinical spectrum, immunopathogenesis, and epidemiology of BCG-related complications, highlighting the impact of vaccine substrain, administration technique, and host immune status on adverse-event rates. On the basis of this evidence, we propose a practical, evidence-based risk-assessment checklist (BCG-RAKE) to support safer vaccine deployment while preserving the substantial TB-control benefits of universal BCG immunization.

## 1. Introduction

Bacille Calmette–Guérin (BCG) remains the only vaccine licensed for the prevention of tuberculosis (TB). It is administered to over 100 million neonates annually [1]. Meta-analyses indicate that BCG confers approximately 50% efficacy against pulmonary TB in adults. However, its protection against tuberculous meningitis and miliary disease in infants approaches 80%. This benefit underpins its continued use in TB-endemic regions [2]. Consequently, the World Health Organization (WHO) recommends a single intradermal dose at birth, except for children with symptomatic HIV infection or confirmed immunodeficiency [3].

Although BCG is generally safe, its live-attenuated nature entails a finite risk of adverse events. These range from self-limited local reactions to life-threatening disseminated infection [4]. The global incidence of BCGosis was estimated at 0.03–0.06 cases per 100,000 doses in early cohorts [5]. Molecular epidemiology studies suggest that the rate may be 100–1000-fold higher in infants with undisclosed severe combined immunodeficiency (SCID) or defects in the interferon-γ/interleukin-12 (IFN-γ/IL-12) axis. The occurrence and severity of complications are influenced by a complex interplay of host factors, notably immune status, and vaccine-related factors, including the specific BCG substrain used [6].

Accurate distinction between “expected” vaccine reactions and true BCG-associated diseases is critical for clinicians and public-health practitioners. Normal reactions include a vesicular stage at the injection site followed by ulceration and scar formation within 2–6 weeks [7]. In contrast, BCG-related diseases comprise suppurative lymphadenitis, osteitis, and BCGosis. Each is associated with distinct genetic risk factors and management algorithms [8]. Recent comparative genomic studies have also linked specific BCG substrains to higher osteitis rates. This highlights the importance of strain selection in national immunisation programmes [9].

Despite these advances, a comprehensive synthesis of incidence data since 2010, stratified by host immune status and vaccine substrain, is lacking. Moreover, no universally adopted screening tool exists to identify high-risk neonates before vaccination. In this systematic review, we collate global evidence on the clinical spectrum, immunopathogenesis, and epidemiology of BCG-related diseases. We also propose a practical, evidence-based risk assessment checklist (BCG-RAKE) to support safer vaccine deployment while maintaining TB control benefits.

## 2. The Clinical Spectrum of BCG-Associated Diseases

Following intradermal BCG vaccination, a cascade of local immunological events is initiated. In the vast majority of healthy recipients, this results in a protective immune response without severe sequelae [10]. However, a spectrum of complications can arise, ranging from common, self-limiting local reactions to severe, systemic diseases [11]. Understanding this clinical spectrum is crucial for accurate diagnosis, appropriate management, and setting patient expectations. Table 1 provides a concise summary of the key characteristics, timing, and risk factors for the main BCG-associated diseases discussed in this section, serving as a quick reference for clinicians.

### 2.1. Normal Vaccination Response and Minor Local Reactions

The expected course post-vaccination is itself a manifestation of a controlled local infection [12]. Typically, within 2–4 weeks, a small, red papule appears at the injection site. This papule gradually softens, becomes a pustule, and may ulcerate, forming a shallow sore approximately 4–10 mm in diameter [13]. This ulcer heals over several weeks to months, leaving a flat or slightly raised, characteristic scar. Mild tenderness and ipsilateral axillary or cervical lymph node enlargement (<1.5 cm) can accompany this process and are considered part of the normal immunogenic response [13,14]. These reactions require no intervention other than routine hygiene and parental reassurance.

### 2.2. Local Complications

#### 2.2.1. Suppurative Lymphadenitis

Suppurative lymphadenitis represents the most frequent significant adverse event associated with BCG vaccination. It is defined by the significant enlargement (usually >1.5–3 cm) and subsequent suppuration of regional lymph nodes—most commonly ipsilateral axillary or cervical nodes—without systemic symptoms [15]. The onset typically occurs 2–6 months post-vaccination. The affected node becomes fluctuant and may fistulize, draining caseous material [16]. The reported incidence varies considerably, influenced by factors such as the BCG strain, vaccine dose, and the skill of the healthcare worker administering the injection.

#### 2.2.2. Cutaneous Complications

A variety of skin manifestations beyond the typical ulcer have been reported, albeit less commonly. These include [17]: BCG lupitis, a rare, chronic granulomatous skin reaction at the vaccination site that resembles lupus vulgaris, presenting as reddish-brown plaques months to years after vaccination. It represents an indolent, local infection requiring antimycobacterial therapy. Hypersensitivity Reactions, these include erythema nodosum and granuloma annulare, which are thought to be immune mediated rather than direct results of infection. Keloid Formation, an abnormal proliferative scar response at the vaccination site, more frequently observed in adolescents and individuals with a genetic predisposition [18].

### 2.3. Regional and Systemic Disease

#### 2.3.1. Osteitis/Osteomyelitis

BCG osteitis is a rare, delayed complication that often presents insidiously, typically 6–24 months after vaccination [19,20]. It most commonly affects the metaphyses of long bones and, less frequently, the sternum or ribs. Clinical signs are often subtle and can include mild swelling, reduced limb movement, or a limp, frequently leading to a significant delay in diagnosis. Radiological findings can be mistaken for pyogenic osteomyelitis or bone tumors [21]. The incidence shows remarkable geographical variation, with higher rates reported in Scandinavian countries compared to other regions, a phenomenon attributed in part to the specific BCG strain used.

#### 2.3.2. Disseminated BCG Disease (BCGosis)

Disseminated BCG disease is the most severe and life-threatening complication [22]. It is defined as a systemic infection caused by the BCG strain, involving one or more non-contiguous sites beyond the regional lymph nodes [23]. The clinical presentation is similar to disseminated TB (miliary TB) and can include prolonged fever, weight loss, hepatosplenomegaly, pancytopenia, and respiratory distress. The onset usually occurs within the first year of life [22,23].

Crucially, disseminated BCGosis is not a random event but is almost exclusively a marker of severe underlying immunodeficiency. It occurs with a very low incidence but carries a historically high mortality rate if undiagnosed and untreated [24]. Prompt initiation of multidrug antimycobacterial therapy is lifesaving. The occurrence of BCGosis should immediately trigger an intensive diagnostic workup for an underlying innate error of immunity (IEI).

## 3. Epidemiology and Risk Factors

The occurrence of BCG-associated adverse events is a multifactorial process, influenced by a complex interplay between host-specific factors, vaccine-related characteristics, and technical aspects of administration [25]. A comprehensive understanding of these determinants is essential for accurately assessing individual risk, implementing effective prevention strategies, and interpreting the wide geographical variation in reported incidence rates [26]. The reported incidence of BCG-associated adverse events shows significant geographical variation, largely attributable to differences in the specific BCG substrains employed in national immunization programs, the prevalence of underlying host immunodeficiencies (e.g., HIV, SCID), and the implementation of public health interventions like newborn screening. A synthesis of recent data stratified by these factors is provided in the fllowing table, offering a clearer perspective on the global landscape.

### 3.1. Host-Related Risk Factors

Overwhelmingly, the most significant risk factor for severe BCG disease is an underlying immunodeficiency in the host that impairs the cell-mediated immune response essential for controlling mycobacterial infections [27].

#### 3.1.1. Inborn Errors of Immunity (IEI)

BCGosis is a well-described and often presenting feature of several IEIs [28]. Its occurrence should be considered a red flag for an underlying immunological defect [14].

Severe Combined Immunodeficiency (SCID), infants with SCID are profoundly lymphopenic and lack functional T cells, which are crucial for containing BCG. Vaccination in these infants frequently leads to rapid, uncontrolled dissemination with high mortality. The incidence of BCGosis in untreated SCID patients is exceptionally high, making BCG vaccination an absolute contraindication [29].

Mendelian Susceptibility to Mycobacterial Disease (MSMD), this group of disorders is characterized by a narrow susceptibility to weakly virulent mycobacteria, including BCG. Mutations in genes involved in the IFN-γ/IL-12 pathway are common causes. These defects impair macrophage activation and the Th1 response, preventing the eradication of intracellular bacteria [30].

Chronic Granulomatous Disease (CGD), patients with CGD have defective phagocyte NADPH oxidase function, preventing the generation of reactive oxygen species needed to kill phagocytosed bacteria [31]. They are highly susceptible to BCG and other catalase-positive organisms, often developing severe local complications or disseminated disease [32].

#### 3.1.2. Acquired Immunodeficiency

HIV Infection, the risk of BCG-related complications is significantly elevated in infants with HIV infection [33]. The degree of immunosuppression, as measured by CD4+ T-cell count, is a critical determinant [34]. The WHO strongly advises against BCG vaccination in children who are known to be HIV-positive and show clinical signs of immunodeficiency due to the high risk of disseminated disease [35]. For infants born to HIV-positive mothers whose status is unknown, revised guidelines recommend vaccination if the infant is asymptomatic, with close monitoring.

Other States of Immunosuppression, iatrogenic immunosuppression is also a contraindication to live vaccination, though this is less relevant in the neonatal period when BCG is typically administered [36].

### 3.2. Vaccine-Related Factors

#### 3.2.1. BCG Strain Variability

Different substrains of BCG, which have evolved during decades of passaging in various laboratories, exhibit genotypic and phenotypic differences in residual virulence and immunogenicity [4]. These differences directly impact reactogenicity and the rate of adverse events [37].

High-Reactogenic Strains, strains such as the Pasteur 1173 P2 and the Danish 1331 have been associated with higher rates of lymphadenitis in some studies [38].

Low-Reactogenic Strains, strains like the Glaxo 1077 (derived from the Danish strain) and the Tokyo 172 have generally been associated with lower rates of local complications [39]. The selection of a specific strain by a national immunization program is a key driver of the country-specific background rate of adverse events.

#### 3.2.2. Dose and Viability

Higher-than-recommended doses, whether due to manufacturing miscalibration or administration error, can overwhelm the immune system and increase the likelihood of complications [40]. Similarly, the use of a vaccine with unexpectedly high viability can have the same effect.

### 3.3. Technical and Operational Factors

Injection Technique, the WHO-recommended route is strictly intradermal. Improper technique, such as subcutaneous or intramuscular injection, can lead to deeper abscess formation [41], increased risk of suppurative lymphadenitis, and a more systemic absorption of the bacilli.

Age at Vaccination, while the risk of disseminated disease is highest in immunodeficient infants, the risk of suppurative lymphadenitis is inversely correlated with age [42]. Neonates and young infants experience the highest rates, likely due to the immaturity of their immune systems.

Table 2 categorizes these risk factors and links them to specific adverse events and preventive actions, providing a structured overview for risk assessment.

The epidemiological data on BCG complications must be interpreted through the lens of these interacting factors. The low overall incidence of severe events, particularly BCGosis, is a testament to the general effectiveness of the human immune system in controlling the vaccine strain [43]. The concentration of serious disease in identifiable high-risk groups provides a clear pathway for risk mitigation through targeted screening and education. Table 3 illustrates the striking geographical variation in complication rates, which can be largely attributed to the factors outlined in Table 2.

**Table 3:** Incidence of BCG-Associated Complications by WHO Region and Select BCG Strains.

*WHO Region/Country*	*BCG Strain Used*	*Suppurative Lymphadenitis (%)*	*Osteitis (Per 100,000)*	*Disseminated BCGosis (Per Million)*	*Primary Notes*
***European Region***					
*S* *candinavia*	Gothenburg/Danish 1331	0.5–2.0	20–40	0.5–1.0	High osteitis rates linked to strain
*United Kingdom*	BCG Danish 1331	0.2–1.5	<1	0.1–0.5	Selective vaccination policy
***Western Pacific***					
*Japan*	Tokyo 172	0.1–0.5	0.1–0.5	0.1–0.3	Lower reactogenicity profile
*South Korea*	Tokyo 172	0.2–0.8	0.2–0.8	0.2–0.6	
***South-East Asia***					
*India*	Russian BCG-I, Danish 1331	0.5–2.0	<1	0.5–2.0	Higher in settings without NBS for SCID
*Thailand*	Tokyo 172	0.3–1.0	<1	0.3–1.0	
***African Region***					
*South Africa*	BCG Danish 1331	0.5–2.0	<1	1.0–5.0	Impact of HIV co-infection
*Kenya*	Russian BCG-I	0.5–2.5	<1	1.0–4.0	
***Region of the Americas***					
*Brazil*	Moreau RDJ	0.2–1.0	<1	0.2–1.0	Universal vaccination
*Argentina*	Danish 1331	0.3–1.2	<1	0.3–1.2	

Note: Incidence data are synthesized from literature published since 2010. Ranges reflect variability within studies and reporting systems.

## 4. Immunopathogenesis

**Figure 1:**
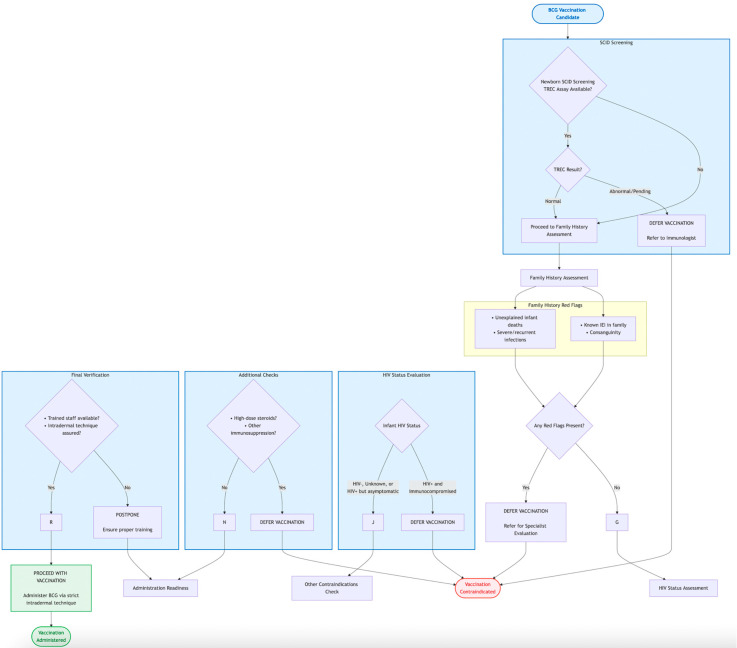
BCG-RAKE (BCG Risk Assessment Checklist) Flowchart for Pre-Vaccination Screening.

The pivotal distinction between a successful immunization and a pathological adverse event following BCG vaccination lies in the host’s ability to mount a robust yet precisely controlled immune response that effectively contains the vaccine strain [44]. The immunopathogenesis of BCG-associated diseases is, therefore, not a story of hyper-virulence of the bacillus, but rather one of a critical failure in host defense mechanisms [45]. This failure disrupts the delicate balance between bacterial containment and elimination, leading to uncontrolled replication and dissemination. Figure 1 provides a stepwise algorithm for the pre-vaccination screening of infants using the BCG-RAKE tool. It guides healthcare workers through the assessment of newborn screening status, family history, HIV status, clinical condition, and administration readiness to determine the suitability for BCG vaccination, operationalizing the concepts discussed in Section 6.

### 4.1. The Normal Immune Response to BCG: A Model of Successful Containment

Following intradermal inoculation, BCG bacilli are phagocytosed primarily by resident macrophages and dendritic cells (DCs) at the injection site. This initial interaction triggers the innate immune system through the engagement of pattern recognition receptors (PRRs), such as Toll-like receptors (TLRs), leading to the production of pro-inflammatory cytokines [46,47].

Antigen-loaded DCs migrate to the draining lymph nodes, where they prime naïve T cells. This event is critical for the initiation of the adaptive immune response. CD4+ T cells differentiate into T-helper 1 (Th1) cells, which are the cornerstone of anti-mycobacterial immunity [48]. These Th1 cells secrete IFN-γ, a key cytokine that potently activates macrophages, enhancing their ability to kill intracellular bacilli through mechanisms such as induction of reactive nitrogen and oxygen species, phagolysosomal fusion, and autophagy [49].

Concurrently, CD8+ T cells are activated and contribute to immunity by lysing infected cells and secreting antimicrobial cytokines. This coordinated response leads to the formation of organized granulomas—the hallmark histological structure of mycobacterial containment [50]. The granuloma, a compact aggregate of macrophages, lymphocytes, and other immune cells, serves to “wall off” the infection, preventing bacterial spread while creating a microenvironment for immune cell collaboration [51]. In immunocompetent individuals, this process results in the eventual clearance of most BCG bacilli. It leaves behind a population of long-lived memory T cells that provide protection against future Mtb challenges, and a localized scar at the injection site [52].

### 4.2. Trained Immunity and Epigenetic Reprogramming

BCG vaccination not only induces specific anti-tuberculosis immunity but also triggers ‘trained immunity’—a phenomenon wherein innate immune cells exhibit enhanced responsiveness to subsequent heterologous challenges following initial antigen exposure. This process is mediated through epigenetic reprogramming, leading to long-term functional reprogramming of innate immune cells.

A recent large-scale multi-omics study published in Immunity (Moorlag et al., 2024) provided comprehensive insights into the individual variation and epigenetic basis of BCG-induced trained immunity [4]. The study systematically profiled 323 healthy volunteers over a 90-day BCG vaccination period, integrating immunological phenotyping, chromatin accessibility (ATAC-seq), and genotyping data.

Key findings revealed that BCG-induced trained immunity is most effective in individuals with a dormant immune state at baseline, characterized by lower innate immune activity and associated chromatin accessibility profiles. In contrast, individuals exhibiting a highly vigilant immune state at baseline showed minimal trained immunity responses. This differential response was closely linked to BCG-induced chromatin remodeling: durable changes in chromatin accessibility were observed at day 90 post-vaccination in trained immunity responders, particularly in genomic regions regulating metabolic reprogramming (e.g., mTOR signaling, glycolysis) and immune-related genes (e.g., *GABBR1*, *PIP4K2B*, *SLC25A1*).

Notably, baseline chromatin accessibility profiles strongly predicted individual capacity to develop trained immunity following BCG vaccination. Machine learning models demonstrated that epigenetic features provided superior predictive power (AUROC = 0.76) for classifying trained immunity responders compared to host factors or seasonal variables alone.

These results indicate that BCG vaccination enhances immune vigilance specifically through epigenetic activation in individuals with dormant immune states, rather than uniformly boosting immunity across all recipients. These findings offer novel perspectives for understanding BCG’s heterologous protective effects and establish a foundation for developing epigenetically informed strategies for personalized immune modulation.

### 4.3. Mechanisms of Pathogenesis: The Breakdown of Containment

In individuals with specific immunodeficiencies, one or more steps in this elaborate defense cascade are compromised, allowing BCG to replicate unchecked.

#### 4.3.1. Defective Granuloma Formation and Function

In conditions like Chronic Granulomatous Disease (CGD), phagocytes cannot generate the oxidative burst required for optimal microbial killing. While granulomas may form, they are often large and necrotic, filled with viable bacteria that persist and can eventually disseminate. The inability to kill the phagocytosed bacilli is the core defect [53].

#### 4.3.2. Impaired T Cell and Macrophage Activation

This is the central defect in Mendelian Susceptibility to Mycobacterial Disease (MSMD) and Severe Combined Immunodeficiency (SCID).

SCID, the near-complete absence of functional T lymphocytes means there is no adaptive immune response. There is no T-cell priming, no IFN-γ production, and no granuloma formation [54]. Macrophages ingest BCG but remain inactivated, becoming mere breeding grounds for the bacilli, which subsequently spread hematogenously [23,55].

MSMD, mutations in the IFN-γ/IL-12 axis disrupt the crucial dialog between innate and adaptive immunity. Macrophages produce IL-12, but T cells cannot respond to it, or T cells produce IFN-γ, but macrophages cannot sense it. In both scenarios, macrophage activation is profoundly impaired [56]. Granulomas may form but are disorganized and ineffective at bacterial control, leading to disseminated infection with BCG or environmental mycobacteria [57].

#### 4.3.3. The Role of “Atonic” or “Overactive” Immunity

In some cases, pathology may arise from an aberrant immune response rather than pure immunodeficiency. An excessive or dysregulated inflammatory response to BCG can contribute to tissue damage, such as the necrosis seen in severe lymphadenitis or the destructive lesions of osteitis [58]. This may involve an overproduction of pro-inflammatory cytokines like TNF-α or an imbalance in immune regulator pathways.

**Figure 2:**
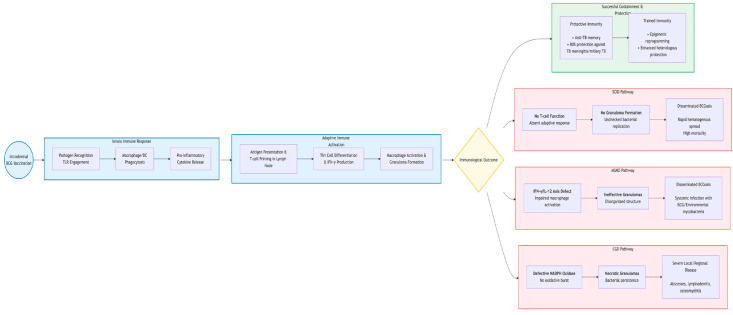
Key Immunological Pathways and Defects Leading to Severe BCG Disease.

In summary, the clinical manifestations of BCG-associated disease are a direct reflection of the underlying immunological defect [59]. Localized complications may result from a partially effective but overly robust inflammatory response, while disseminated disease is unequivocally the consequence of a severe failure in the core mechanisms of cell-mediated immunity required to control intracellular pathogens [60]. This understanding is paramount for diagnosing the underlying condition and for developing targeted therapeutic strategies, such as recombinant IFN-γ therapy in some MSMD patients [61]. Figure 2 illustrates the immunopathogenic cascade leading to BCGosis, directly linking to the mechanisms described in this section. It highlights the critical immune pathways involved in controlling BCG (e.g., IFN-γ/IL-12 axis, phagocyte oxidative burst, T-cell activation) and visually maps the specific defects in SCID, MSMD, and CGD that result in the failure of bacterial containment and subsequent disseminated disease.

## 5. Diagnosis, Treatment and Management

The management of BCG-associated diseases presents unique challenges, necessitating a high index of suspicion, prompt and accurate diagnostic confirmation, and tailored therapeutic strategies. The approach varies significantly based on the severity of the complication, underscoring the importance of a structured and evidence-based algorithm for clinicians.

### 5.1. Diagnostic Challenges and Approach

A key diagnostic challenge lies in differentiating BCG-related disease from infections caused by *Mycobacterium tuberculosis* (Mtb) or non-tuberculous mycobacteria (NTM), as the clinical and histological presentation can be indistinguishable [62,63].

#### 5.1.1. Clinical and Histopathological Clues

A history of BCG vaccination, particularly within the last 1–2 years, is the primary clue. The timing of symptom onset can suggest specific entities [64]. Biopsy specimens from affected sites typically show granulomatous inflammation with caseous necrosis, identical to that seen in TB. The presence of acid-fast bacilli (AFB) on staining confirms a mycobacterial etiology but does not identify the species [65].

#### 5.1.2. Microbiological Confirmation: The Role of Molecular Tools

Definitive diagnosis requires microbiological identification of the BCG strain. This is critical to avoid misdiagnosis as wild-type TB, which would lead to unnecessary prolonged treatment and contact tracing [66].

Isolation of the mycobacterium in culture is the traditional gold standard. BCG can be distinguished from Mtb by its specific biochemical characteristics and susceptibility to pyrazinamide (PZA) in vitro [67].

Nucleic Acid Amplification Tests (NAATs), these are now the cornerstone of rapid diagnostics. PCR-based assays can rapidly identify members of the *M. tuberculosis* complex (MTBC). Crucially, specific genetic markers can differentiate BCG from wild-type Mtb [68]. The most reliable target is the RD1 region, which is absent in all BCG strains but present in all Mtb strains. Detection of RD1 confirms Mtb, while its absence in a positive MTBC sample is highly indicative of BCG [69].

Whole-Genome Sequencing (WGS), while not routine, WGS provides the most definitive speciation and can be used in complex or atypical cases.

### 5.2. Treatment Strategies

Treatment should be tailored to the type and severity of BCG-related complications. Localized suppurative lymphadenitis can often be managed conservatively when nodes are small and non-fluctuant, as many resolve spontaneously; however, large or fluctuant masses are best treated by repeated needle aspiration, which both relieves symptoms and yields diagnostic material [70].

When lesions are large, progressive, or draining, a 3–6-month course of isoniazid and rifampicin is usually recommended, although no universal regimen exists. Surgical excision is reserved for cases that fail both aspiration and chemotherapy, whereas incision and drainage are discouraged because of the high risk of chronic sinus formation [71].

BCGosis constitutes a medical emergency requiring immediate empirical multidrug therapy. Because BCG is intrinsically resistant to pyrazinamide, standard treatment consists of isoniazid and rifampicin supplemented by a fluoroquinolone, ethambutol, or a macrolide for 9–12 months, with duration adjusted according to clinical response and, when possible, susceptibility testing [72].

Adjunctive measures include surgical biopsy or drainage of large abscesses, recombinant interferon-γ for patients with identified IFN-γ/IL-12 pathway defects, and granulocyte colony-stimulating factor in chronic granulomatous disease to enhance neutrophil function. For individuals with underlying SCID, the only curative option is hematopoietic stem-cell transplantation, which must be performed under optimal antimycobacterial coverage to prevent fatal reactivation during immunosuppressive conditioning [73].

### 5.3. Adverse Event Monitoring and Reporting

Robust pharmacovigilance systems are essential for understanding the true epidemiology of BCG complications. All suspected serious adverse events following immunization (AEFI) should be reported to national health authorities. This data is vital for informing vaccine policy, assessing the safety profile of different BCG strains, and identifying potential shifts in risk patterns [74]. Table 4 summarizes the recommended treatment approaches for the main BCG-associated diseases, providing a clear guide for clinicians based on disease severity and presentation.

A high standard of clinical care, coupled with accurate diagnostics and a low threshold for investigating immunodeficiency, is paramount for the successful management of these rare but serious vaccine-related events.

## 6. Risk–Benefit Assessment and Prevention Strategies

The deployment of any medical intervention, particularly a vaccine used on a global scale, necessitates a continuous and rigorous evaluation of its risks against its benefits. For the BCG vaccine, this balance is stark yet unequivocally tilted in favor of its use in high-burden settings. The core of modern public health strategy lies not in questioning its overall value, but in implementing sophisticated, evidence-based measures to identify the small subset of individuals for whom the risk is unacceptably high, thereby maximizing population-level benefit while minimizing individual harm [75].

### 6.1. Global Perspective on Risk–Benefit Analysis

The calculus of BCG vaccination is profoundly influenced by the local epidemiology of TB. In high TB-burden countries, the lifetime risk of a child contracting TB and developing its severe forms is substantial. Here, the benefit of BCG is immense, meta-analyses confirm that a single dose of BCG at birth reduces the risk of tuberculous meningitis and miliary TB by approximately 70–80%. This translates to the prevention of tens of thousands of childhood deaths and cases of severe disability annually [76]. In contrast, the risk of severe adverse events is vanishingly small. The incidence of disseminated BCGosis is estimated at less than 1 per million doses, while localized lymphadenitis, though more common (0.1–1%), is typically manageable and non-fatal [77].

Therefore, from a public health perspective, the number of severe TB cases and deaths prevented by BCG vaccination dwarfs the number of severe adverse events caused by it. This favorable ratio justifies the WHO’s recommendation for universal BCG vaccination of neonates in high-burden countries.

The equation shifts in low TB-burden countries. The risk of a child developing severe TB is very low, making the absolute benefit of BCG smaller. Consequently, the same low absolute risk of vaccine-related complications becomes more significant in the risk–benefit analysis [78]. This is why many such countries have adopted selective vaccination policies, targeting only high-risk groups. This stratified approach optimizes the benefit-risk profile for their specific epidemiological context [79].

### 6.2. Core Prevention Strategy

The most effective strategy to prevent severe BCG-associated disease is to avoid administering the vaccine to individuals with known contraindications. This hinges on effective pre-vaccination screening. A multi-pronged approach is essential, combining a low-cost, immediately implementable family history review with advanced screening technologies where available. Table 5 summarizes the core components of this preventive strategy.

#### 6.2.1. Medical History Assessment

Before vaccination, a comprehensive family and personal medical history should be obtained as the first and most accessible line of defense. This is a cost-effective and immediately implementable strategy, particularly crucial in regions where universal newborn screening for SCID is not yet feasible due to economic and infrastructural challenges. Vaccination should be postponed pending further evaluation if any of the following red flags are present: a history of severe, unusual, or recurrent infections in the individual or family; infant deaths in the family possibly linked to infection; a known congenital immunodeficiency in any family member; a history of parental consanguinity; or confirmed or suspected HIV infection in the mother or infant [80].

#### 6.2.2. The Pivotal Role of Newborn Screening (NBS) for Severe Combined Immunodeficiency (SCID)

The TREC assay, performed on a routine newborn blood spot, identifies infants with profoundly low T-cell lymphopenia, a hallmark of SCID. The implementation of population-based NBS for SCID using the T-cell receptor excision circle (TREC) assay represents a transformative advancement in preventive vaccinology [81]. By providing a diagnosis within the first days of life—before BCG vaccination is typically administered—NBS allows clinicians to definitively withhold BCG from these most vulnerable infants. This single intervention can virtually eliminate BCGosis as a presenting feature of SCID, drastically improving the chances of successful treatment for the underlying immunodeficiency [82].

The integration of SCID screening into national NBS programs is the most powerful and cost-effective strategy for preventing fatal disseminated BCG disease and should be a public health priority in all countries utilizing universal BCG vaccination.

#### 6.2.3. HIV Status Determination

Adherence to WHO guidelines is critical: BCG is contraindicated in infants who are HIV-positive and show clinical signs of immunodeficiency. For infants born to HIV-positive mothers, vaccination can proceed if the infant is asymptomatic and its HIV status is not yet known, with close follow-up [83]. Rapid diagnostic testing for HIV in the perinatal period is essential for making this decision.

#### 6.2.4. The BCG-RAKE Checklist

Based on the synthesized evidence, we propose the BCG-RAKE (BCG Risk Assessment Knowledge and Evaluation) checklist as a practical tool for pre-vaccination risk assessment. Its core components are summarized in Table 6, which can be integrated into routine immunization workflows to standardize screening and reduce human error. The checklist is designed to be applicable across diverse healthcare settings, from well-resourced hospitals with established newborn screening programs to primary care clinics in resource-limited areas. The logical flow of the screening process is also depicted in Figure 1 (Section 4), providing both a detailed tabular and an easy-to-follow visual guide for implementation.

### 6.3. Future Directions: Next-Generation Vaccines

The ultimate solution to the risk of BCG-associated adverse events lies in the development of safer, more effective TB vaccines that can potentially replace BCG [84]. The vaccine pipeline includes candidates such as subunit vaccines (e.g., M72/AS01E), viral-vectored vaccines, and whole-cell mycobacterial vaccines. Among the most promising are mRNA-based TB vaccines, which offer a highly adaptable and potentially safer platform as they do not involve a live organism [85]. While these candidates are still under evaluation in clinical trials, their successful development could one day eliminate the risk of BCG-related disease entirely while providing superior protection against all forms of TB [86]. Recent phase 2b trial results for the M72/AS01E vaccine candidate showed approximately 50% efficacy in preventing pulmonary TB in adults, demonstrating the feasibility of a post-BCG booster. However, challenges remain, including the need for broader efficacy across age groups and in HIV-positive populations, ensuring stability in resource-limited settings, and navigating complex regulatory pathways. The ongoing research and investment in these next-generation platforms are crucial for ultimately achieving TB control goals without the risk of live vaccine-associated complications.

In conclusion, the benefits of BCG vaccination in TB-endemic regions are profound and indisputable. The risk of severe adverse events is low and can be further minimized through the systematic implementation of preventive strategies, most notably universal newborn screening for SCID, the rigorous application of a detailed family history review, and the use of standardized pre-vaccination checklists like BCG-RAKE. A vigilant, multi-pronged approach ensures that this cornerstone of global TB control remains as safe as possible for every child.

## 7. Conclusions and Future Perspectives

The BCG vaccine remains an indispensable tool in the global fight against tuberculosis, particularly for the prevention of life-threatening forms of the disease in children. This review has synthesized the current understanding of the clinical spectrum, immunological mechanisms, and risk factors associated with BCG-related complications. The central theme that emerges is one of balance: a delicate equilibrium between the potent, life-saving immunogenicity of the vaccine and the rare but serious consequences that arise when host defenses fail to contain the live-attenuated strain.

The central conclusion that emerges is that the overwhelming public health benefit of BCG vaccination in TB-endemic regions justifies its continued use. The prevention of childhood tuberculous meningitis and miliary TB far outweighs the low incidence of severe adverse events. Crucially, the most severe of these events, disseminated BCGosis, is not a random tragedy but a predictable consequence of severe underlying immunodeficiency. The risk is therefore not distributed equally across the population but is concentrated in a small, identifiable subset of infants. This understanding reframes the problem from one of vaccine safety to one of precision public health: the goal is not to discard this vital tool but to implement strategies to prevent its administration to those for whom the risk is unacceptably high.

The primary strategy for risk mitigation is unequivocal: rigorous pre-vaccination screening. This includes a careful family history and, most importantly, the integration of newborn screening (NBS) for Severe Combined Immunodeficiency (SCID) using the TREC assay into national immunization programs. Tools like the BCG-RAKE checklist (Table 6 and Figure 1) operationalize this screening process, making it actionable for frontline healthcare workers. Furthermore, adherence to contraindications, particularly regarding symptomatic HIV infection, and ensuring proper vaccination technique are essential components of a comprehensive safety framework.

Looking to the future, several key avenues will shape the management of BCG-related risks and the broader fight against TB:

1. Global Expansion of Newborn Screening: A major public health effort is needed to make SCID screening accessible and routine in all countries that employ universal BCG vaccination. This will require overcoming significant economic and infrastructural barriers, especially in low- and middle-income countries (LMICs) but promises to virtually eliminate the deadliest complication of the vaccine. Strengthened Pre-Vaccination Screening: In the immediate term, enhancing the systematic use of detailed family history questionnaires, as operationalized by the BCG-RAKE checklist, provides a viable and critical strategy to identify at-risk infants in all resource settings.
2. Advancements in Diagnostic Tools: The development and deployment of rapid, low-cost molecular point-of-care tests to differentiate BCG from Mtb complex will expedite diagnosis and ensure appropriate management of adverse events, preventing unnecessary anti-TB treatment.
3. The Pursuit of a Safer, More Effective Successor: The long-term solution lies in the development and deployment of next-generation TB vaccines. Promising candidates, such as protein-adjuvant vaccines (e.g., M72/AS01E) or recombinant viral vectors, offer the potential for superior efficacy against pulmonary TB in adults without the risk of dissemination associated with live vaccines. The gradual replacement of BCG with a safer and more effective alternative remains the ultimate goal of TB vaccinology.
4. Deepening Immunological Understanding: Ongoing research into the human genetics of mycobacterial susceptibility will continue to uncover novel immunodeficiency syndromes, further refining our understanding of risk and allowing for ever-more precise screening protocols.

In summary, BCG-associated disease is a rare but serious consequence of a vaccine that remains indispensable in the global fight against tuberculosis. Through a combination of vigilant clinical practice, the strategic application of screening technologies, and continued support for vaccine research, we can continue to harness the life-saving benefits of BCG while protecting the most vulnerable among us. This balanced, evidence-based approach is essential until the day when newer, safer vaccines finally consign both TB and the complications of its prevention to the pages of history.

## Figures and Tables

**Table 1:** Summary of Key BCG-Associated Diseases.

*Clinical Entity*	*Typical Onset*	*Key Features*	*Estimated Incidence*	*Primary Risk Factor*
*Suppurative Lymphadenitis*	2–6 months	Fluctuant, ipsilateral lymph node (>1.5 cm), may fistulize	0.1–1%	Infant age, vaccine strain, injection technique
*Osteitis/Osteomyelitis*	6–24 months	Insidious onset, metaphysis of long bones, limp	0.1–40/100,000	Specific BCG strain (e.g., Gothenburg)
*Disseminated BCGosis*	3–12 months	Systemic illness (fever, hepatosplenomegaly, pancytopenia)	0.1–1.0/1,000,000	Underlying severe immunodeficiency

Note: Incidence rates are approximate and vary by region and BCG strain. BCGosis, disseminated BCG disease.

**Table 2:** Key Risk Factors for BCG-Associated Diseases.

*Risk Factor Category*	*Specific Factor*	*Associated Adverse Event(s)*	*Preventive Action*
***Host-Related***	SCID, MSMD, CGD	BCGosis	Absolute contraindication. Pre-vaccination screening (family history, NBS for SCID).
	HIV/AIDS (symptomatic, immunocompromised)	Disseminated BCGosis, localized complications	Absolute contraindication. HIV testing of infant/mother prior to vaccination.
***Vaccine-Related***	High-reactogenicity strain	Suppurative lymphadenitis	National program strain selection based on benefit-risk profile.
	Overdose/High potency	Local and systemic complications	Adherence to good manufacturing and distribution practices.
***Technical***	Subcutaneous/intramuscular injection	Abscess, severe lymphadenitis	Strict intradermal technique. Training of healthcare workers.
	Young infant age (<3 months)	Suppurative lymphadenitis	Adherence to recommended immunization schedule

Note: NBS, newborn screening; SCID, severe combined immunodeficiency; MSMD, Mendelian Susceptibility to Mycobacterial Disease; CGD, Chronic Granulomatous Disease.

**Table 4:** Summary of Treatment Recommendations for BCG-Associated Diseases.

*Clinical Condition*	*First-Line Treatment*	*Alternative/Adjunctive Options*	*Duration*	*Key Considerations*
***Suppurative Lymphadenitis***	Aspiration; Observation	INH + RIF	3–6 months (if medical therapy used)	Avoid incision & drainage.
***Osteitis/Osteomyelitis***	INH + RIF + (FQ or EMB)	Surgery for debridement/diagnosis	9–12 months	Ensure PZA is NOT included.
***Disseminated BCGosis***	INH + RIF + FQ + (EMB or Macrolide)	Adjunctive IFN-γ (for MSMD); G-CSF (for CGD); HSCT (for SCID)	≥9–12 months	Treat underlying immunodeficiency

Note: INH, isoniazid; RIF, rifampicin; FQ, fluoroquinolone; EMB, ethambutol; PZA, pyrazinamide; IFN-γ, interferon-gamma; G-CSF, granulocyte colony-stimulating factor; HSCT, hematopoietic stem cell transplantation; MSMD, Mendelian Susceptibility to Mycobacterial Disease; CGD, Chronic Granulomatous Disease; SCID, Severe Combined Immunodeficiency.

**Table 5:** Summary of Key Prevention Strategies for BCG-Associated Disease.

*Strategy*	*Target Group*	*Action*	*Expected Outcome*
***Newborn Screening (TREC assay)***	All newborns in BCG-vaccinating countries	**Defer BCG vaccination** until SCID result is available. If positive, **absolute contraindication**.	Near-elimination of BCGosis in SCID patients.
***HIV Testing***	Infants born to HIV-positive mothers	Determine infant’s HIV status. **Withhold BCG** if infant is HIV+ and immunocompromised.	Prevention of BCGosis in HIV-infected infants.
***Family History Review***	All infants prior to vaccination	**Defer vaccination** if history suggests inherited immunodeficiency; refer for specialist evaluation.	Identification of at-risk infants beyond SCID (e.g., CGD, MSMD).
***Healthcare Worker Training***	Vaccinators	Education on: **strict intradermal technique**; **recognizing contraindications**; **managing minor reactions**.	Reduction in technical errors (e.g., abscesses) and inappropriate administration.

Note: TREC, T-cell receptor excision circle; SCID, severe combined immunodeficiency; CGD, Chronic Granulomatous Disease; MSMD, Mendelian Susceptibility to Mycobacterial Disease.

**Table 6:** The BCG-RAKE (BCG Risk Assessment Knowledge and Evaluation) Checklist.

*Screening Domain*	*Key Screening Questions/Criteria*	*Action Required*
***1. Newborn Screening (NBS) Status***	Is the result of the newborn SCID (TREC) screening available and normal?	If result is **pending**: DEFER vaccination until result available. If result is **abnormal**: ABSOLUTE CONTRAINDICATION. Refer to immunologist.
***2. Family History***	Is there a history of unexplained infant deaths or deaths due to severe infection? Is there a known diagnosis of an Inborn Error of Immunity (IEI) (e.g., SCID, CGD, MSMD) in the family? Is there a history of severe, unusual, or recurrent infections in the infant or family members? Is there a history of parental consanguinity?	If **YES** to any question: DEFER vaccination. Refer for specialist immunological evaluation.
***3. HIV Status***	Is the infant confirmed to be HIV-positive and immunocompromised?	If YES: ABSOLUTE CONTRAINDICATION If born to an HIV-positive mother but asymptomatic and status unknown: VACCINATE with close follow-up.
***4. Clinical Status & Other Contraindications***	Does the infant have a fever or acute illness? Is the infant on high-dose steroids or other significant immunosuppressive therapy? Is there a localized skin infection at the planned injection site?	If **YES** to any question: POSTPONE vaccination until full recovery or therapy is completed.
***5. Administration Readiness***	Is a trained healthcare worker available to administer a **strictly intradermal** injection?	If **NO**: POSTPONE until trained personnel and correct technique are assured.

Note: SCID, severe combined immunodeficiency; TREC, T-cell receptor excision circle; IEI, inborn errors of immunity; CGD, Chronic Granulomatous Disease; MSMD, Mendelian Susceptibility to Mycobacterial Disease.

## Data Availability

The raw data supporting the conclusions of this article will be made available by the authors on request.
